# Development and content validity of the CENA Program for Educational Training on the Neuropsychology of Learning, with an emphasis on executive functions and attention

**DOI:** 10.1590/1980-57642016dn11-010012

**Published:** 2017

**Authors:** Janice R. Pureza, Rochele P. Fonseca

**Affiliations:** 1 Psychologist. PhD Student, Graduate School of Psychology, Pontifical Catholic University of Rio Grande do Sul (PUCRS).; 2 Head of the Clinical and Experimental Neuropsychology Research Group (GNCE), Pontifical Catholic University of Rio Grande do Sul, RS, Brazil.

**Keywords:** executive functions, neuropsychological intervention, education, content validity, school neuropsychology

## Abstract

**Introduction:**

The importance of executive functions (EF) in childhood development, and their role as indicators of health, well-being, professional and academic success have been demonstrated by several studies in the literature. FE are cognitive processes that aim to control and manage behavior to achieve specific goal and included skills planning, inhibition, cognitive flexibility, (executive) attention and the central executive component of working memory (WM). In the context of education, the EF are crucial for continued learning and efficient academic performance due to their involvement in several components of the educational process.

**Objective:**

The aim of this article was to describe the development and content validity of the CENA Program for Educational Training on the Neuropsychology of Learning, with an emphasis on executive functions and attention.

**Methods:**

The study involved seven specialists (four responsible for evaluating the program, and three involved in brainstorming), and was carried out in three stages:

1. Background research: neuropsychology and education;
2. Program development - author brainstorming and
3. Evaluation by expert judges The goals, language and methods.

**Results:**

CENA Program were considered adequate, attesting to its content validity as a school-based neuropsychological intervention.

**Conclusion:**

Teacher training in school neuropsychology may be an important area for future investment and contribute to academic achievement and student development in the Brazilian education system.

## Abstract (pt)

**Introdução:**

Estudos demonstram a importância das funções executivas (FE) para o desenvolvimento infantil, salientando esses processos como indicadores de saúde, bem-estar e sucesso escolar e profissional. As FE são processos cognitivos que objetivam o controle e o gerenciamento do comportamento para atingir metas específicas e envolvem processos como planejamento, automonitoramento, inibição, flexibilidade cognitiva, atenção (executiva) e componente executivo central da memória de trabalho (MT). No que se refere ao contexto escolar, as FE são necessárias para o contínuo de aprendizagem e um eficiente desempenho acadêmico, pois são habilidades cognitivas recrutadas em diversas situações na escola.

**Objetivo:**

Este artigo tem por objetivo apresentar o processo de construção e validade de conteúdo do CENA – Programa de capacitação de educadores sobre neuropsicologia da aprendizagem com ênfase em funções executivas e atenção.

**Métodos:**

Participaram deste estudo sete profissionais especialistas (quatro juízes responsáveis por avaliar o programa e três especialistas participantes de *brainstormings*) e envolveu três etapas distintas:

1. pesquisa a pressupostos teóricos: neuropsicologia e educação;
2. construção do programa e *brainstorming* entre autores e
3. análise de juízes.

**Resultados:**

O CENA mostrou adequação em relação aos seus objetivos, estrutura, linguagem e método, apresentando evidência de validade de conteúdo para um processo de intervenção neuropsicológica em âmbito escolar.

**Conclusão:**

O investimento em programas de capacitação de professores em neuropsicologia escolar pode ser uma contribuição para abordagens que busquem o desenvolvimento acadêmico e um aprimoramento da aprendizagem na educação brasileira.

## INTRODUCTION

The importance of executive functions (EF) in childhood development, and their role as indicators of health, well-being, professional and academic success have been demonstrated by several studies in the literature.^1-4^ The EF are cognitive processes involved in the control and management of goal-oriented behavior. The skills embedded in this construct include organization, planning, self-monitoring, cognitive and behavioral initiation and inhibition, cognitive flexibility, the selection of problem-solving strategies, as well as (executive) attention and the central executive component of working memory (WM).^1,3,5-8^ More specifically, recent approaches highlight that the EF include constructs distinguishable from one another yet intercorrelated.^9,10^

In the context of education, the EF are crucial for continued learning and efficient academic performance due to their involvement in several components of the educational process, such as:

[a] the ability to handle new, unexpected and/or challenging tasks, which require additional concentration for successful completion;
[b] organization and planning skills;
[c] impulse control as well as behavioral and emotional regulation;
[d] the ability to explore varied problem-solving strategies.^11,12^

The EF are also involved with the cognitive skills required for the acquisition of reading, writing and mathematical skills.^13-15^ As a result, executive dysfunction is associated with learning disorders (dyslexia, dyscalculia), and other clinical conditions with a negative impact on school performance (Attention Deficit Hyperactivity Disorder; ADHD),^16-19^ as well as other neurodevelopmental disorders.

These observations highlight the need for public policy regarding the development of educational interventions which move beyond the remediation (assessment, orientation and rehabilitation) of learning difficulties (regardless of associated clinical conditions), cognitive impairments and/or behavioral alterations. Preventive interventions involving the stimulation of the EF have become an increasingly important tool for the promotion of better academic outcomes. The adequate stimulation of EF can optimize the acquisition of reading, writing and mathematical skills.^12,15^

As demonstrated by the current literature, significant investments have been made in interventions targeting the cognitive and emotional skills (self-regulation and metacognition) required for childhood development and learning. These interventions contribute to educational practice, and promote the use of more effective strategies and approaches in the teaching-learning process.^20-22^

This claim is supported by previous studies in which programs targeting the stimulation of socio-emotional and self-regulation skills in the context of learning were found to improve executive processing in schoolchildren. Examples of such interventions include the Head Start REDI program, the Chicago School Readiness Project – CSRP, the Promoting Alternative Thinking Strategies (PATHS) Program and the Tools of Mind intervention in the United States,^23-27^ and the Sarilhos do Amarelo Program in Portugal.^28^ Most of these methods target preschool children, and were designed to be implemented by teachers under the supervision of interdisciplinary teams. In Brazil, similar strategies with a more direct involvement in educational processes have also been implemented. These include the NEUROEDUCA project, which offers teacher training in neuroscience and the management of learning disorders.^21^ Teacher-mediated interventions for the stimulation of the EF include the PIAFEX Intervention Program for Self-regulation and the Executive Functions *(Programa de Intervenção em Autorregulação e Funções Executivas),* developed for preschool children.^29^ This program aims to improve executive functioning by stimulating executive and self-regulation skills.

Unlike the development of assessment instruments, the construction of intervention programs has received little attention in the neuropsychological literature.^30-33^ Nevertheless, it must be ensured that the construction of neuropsychological interventions follows the same strict methodological guidelines as the development of cognitive assessment tools.

In light of previously mentioned issues, including the scarcity of teacher training programs and methodological articles in the field of school neuropsychology, the aim of this study was to describe the construction and content validity of a training program for 2^nd^ and 3^rd^ grade public school teachers, involving the stimulation of EF in children. The intervention developed in this study was called the CENA Program for Educational Training on the Neuropsychology of Learning, with an emphasis on EF and attention *(Programa de capacitação de educadores sobre neuropsicologia da aprendizagem com ênfase em FE e atenção)*.^34^ The program is unique in its attempt to promote teacher autonomy by integrating the stimulation of EF into the professional practice. As a result, it may also be a useful tool for researchers and clinical practitioners seeking to develop educational strategies involving the EF as mediators of learning.

## METHODSW

**Participants.** A team of seven subject specialists took part in the present study. Three were involved in the brainstorming stage while four were responsible for judging whether the program achieved its ultimate objectives. The academic background of each participant and the lengths of their careers in neuropsychology are shown in Table 1. All participants had at least 3 years' experience as neuropsychologists.

**Table 1:** Characteristics of professionals consulted for development of the program.

Specialists judges	Educational training area	Higher educational level	Professional area	Length of experience in the professional area
Judge 1	Psychology	Doctoral degree in progress	Neuropsychology	7 years
Judge 2	Phonoaudiology	Doctoral degree in progress	Phonoaudiology and neuropsychology	4 years
Judge 3	Phonoaudiology	Doctoral degree	Phonoaudiology	9 years
Judge 4	Language arts and psychology	Master's degree	Education	5 years
Specialist 1	Phonoaudiology and psychology	Post-doctoral	Neuropsychology	16 years
Specialist 2	Psychology	Doctoral degree in completion	Neuropsychology	8 years
Specialist 3	Psychology	Doctoral degree in completion	Psychology and neuropsychology	12 y

**Figure 1:**
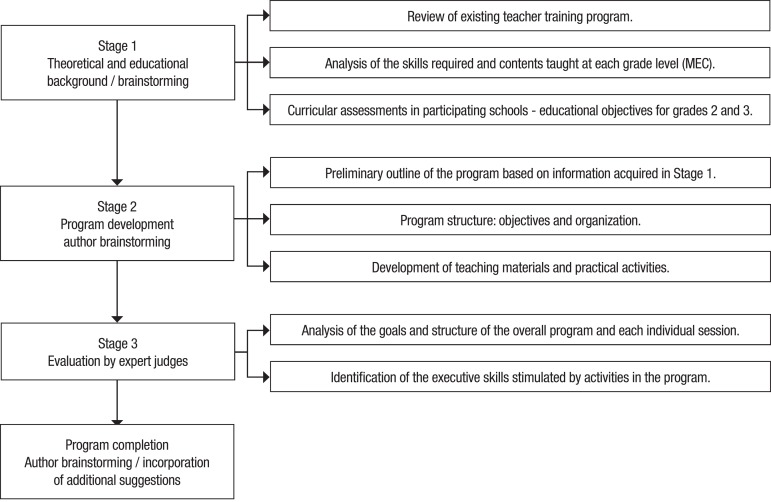
Stages in the construction of the CENA Program.

**Procedures and instruments.** The stages involved in the construction of the CENA Program are displayed in Figure 1. Methodological guidelines for the construction and adaptation of neuropsychological instruments were followed as closely as possible throughout the process.^31-33,35^

1. *Background research: neuropsychology and education.* The scientific and theoretical background of the intervention was constructed based on a review of the literature on teacher training programs. Legal guidelines issued by the Ministry of Education and Culture (MEC) regarding the skills required and the content explored in each school year were then examined.^36^ This procedure was carried out with the following aims: [a] to identify the goals of different stages in primary school education; [b] to identify the cognitive skills required by the teaching and assessment methods employed in different school years; [c] to adapt the techniques and contents of the CENA Program to the school years in question, and to the cognitive-developmental stage of the students at these grades. An analysis of the national curriculum for Portuguese and Mathematics in grades 1 through 4, as determined by the MEC, was performed.^36^ School curricula were then analyzed to verify the educational goals and the resources available to teachers in grades 2 and 3. A brainstorming session was then scheduled with specialists in order to reflect on the findings and make arrangements for the next stage in the development of the intervention.
2. *Program development - author brainstorming.* This stage involved the construction of a preliminary outline for the CENA Program based on the information collected at stage 1. In addition, a brainstorming session about knowledge regarding the types of task used to evaluate different cognitive domains in childhood was conducted with three experts.
  The next step was the development of supporting materials, such as pamphlets describing EF-based teaching strategies, classroom management, and specific instructions regarding the stimulation of the cognitive components targeted by the program. This was done to provide educators with additional knowledge on childhood neurocognitive development, especially with regards to the cognitive skills involved in Portuguese and Mathematics: attention (visual and auditory, focused and alternating), language (phonological, lexical-semantic, syntactic, discourse (narrative) and pragmatic development – at the comprehension and expression levels), memory (short and long term – semantic, episodic and WM), EF (planning, inhibition, cognitive flexibility) and self-regulation. Moreover, strategies were developed to encourage and practice the use of these functions in the classroom using resources that were available to both teachers and students. Some of the tasks were inspired by neuropsychological assessment tools, such as the Wisconsin Card Sorting Test, the Trail Making Test, and the Stroop Color-Word Test, as well as the Go/No-go, n-back and cancellation paradigms, in addition to teaching materials for grades 2 and 3.^37,38^ Activities were planned in two stages: [a] some activities were planned *a priori*, as a general model for the stimulation of the cognitive functions targeted in the program; and [b] other activities were developed *a posteriori*, over the course of the program, in response to teachers' demands. These activities were developed based on previously mentioned sources and underlying cognitive constructs, as well as teachers' evaluations of their classroom applicability. This was a fundamental part of the program, since one of its major aims is to provide teachers with the resources to create and implement their own strategies based on relevant educational and curricular objectives.
  The next step was the organization of the program structure. This involved the development of additional content corresponding to the cognitive skills targeted, and the organization of course materials into a schedule that would be adequate for the teachers enrolled in the program. Before submitting the material to an evaluation by expert judges, a further brainstorming session was scheduled with specialists for final reflections and arrangements.
3. *Evaluation by expert judges.* The CENA was evaluated in two steps, each with a different focus. The first was an assessment of its specific objectives and the extent to which these were covered by the program activities. The second consisted of an evaluation of the executive components stimulated by the activities and strategies planned.
  Inter-rater agreement was examined using the Content Validity Index (CVI), which indicates the percentage of judges who agree on particular aspects of an instrument when analyzed as a whole, and on an individual item basis.^37^ Each rater provides a score on a Likert scale ranging from 1 to 4, corresponding to the extent to which they believe the intended construct is represented by the item/instrument in question (1=not representative; 2=major revision required to achieve representativeness; 3=minor revision required to achieve representativeness; 4=representative). An instrument must have a minimum CVI of 0.90 to be considered representative.^39^

## RESULTS

The results of each stage in the construction process are described below.

**Background research: neuropsychology and education.** The investigation of existing programs showed that the majority of early/preventive interventions focused on EF and self-regulation in pre-school children, emphasizing the development of self-regulation, metacognition, WM, inhibition and cognitive flexibility skills.

The improvement of these abilities facilitates the acquisition of reading, writing and mathematical skills. The guiding principle of these interventions is that the cognitive skills targeted can be taught within the school environment. To achieve this goal, these programs employ a series of strategies and activities targeting each individual executive component. Schoolteachers take on the role of mediators of the psychoeducation process for their students. These programs were used as the basis for an intervention which involved the stimulation of EF in primary school children, but had the teachers themselves as the target population. The literature on interventions for the stimulation of EF in school-age children is summarized in Table 2.

**Table 2:** Intervention programs for stimulation of executive functions in the school environment.

References	Objectives	Target audience	Operationalization
**Executive functions and school readiness intervention: impact, moderation, and mediation in the Head Start REDI program ** Bierman, Nix, Greenberg, Blair & Domitrovich, 2008 (USA)	• To stimulate the development of social and emotional skills in young children.	• Preschool children • Teachers	• Program developed as curriculum to be implemented in the classroom. • Mediated by teachers. • Teachers were monitored and received training during the implementation of the program.
**CSRP's Impact on Low-Income Preschoolers' Preacademic ** **Skills: Self-Regulation as a Mediating Mechanism ** Raver, Jones, Li-Grining, Zhai, Bub & Pressler, 2011 (USA)	• To stimulate school readiness and executive, emotional and behavioral skills in low-income children.	• Preschool children • Teachers	• Program developed as curriculum to be implemented in the classroom. • Mediated by teachers. • Teachers were monitored and received training during the implementation of the program.
**Promoting Alternative Thinking Strategies Program - PATHS ** Greenberg et. al., 1995; Riggs et. al., 2006 (USA)	• To stimulate school readiness and executive, emotional and behavioral skills in low-income children.	• Preschool children • Teachers	• Program developed as curriculum to be implemented in the classroom. • Mediated by teachers. • Teachers were monitored and received training during the implementation of the program.
**Sarilhos do Amarelo** Rosário, Nuñes & Gonzales-Pienda, 2007 (Portugal)	• To stimulate self-regulation skills and create opportunities and strategies to increase learning.	• Children aged 5 to 10 years • Teachers	• Program developed as curriculum to be implemented in the classroom. • Mediated by teachers.
**Tools of Mind** Bodrova & Leong, 2001 (USA)	• To stimulate the growth of basic cognitive skills for literacy.	• Preschool children • Teachers	• Program developed as curriculum to be implemented in the classroom. • Mediated by teachers.
**Programa de Intervenção em Autorregulação e Funções Executivas - PIAFEX** Dias & Seabra, 2013 (Brazil)	• To increase self-regulation and executive functions in the school setting. • To guide education professionals about EF, emotional regulation and metacognition.	• Children aged 5 to 6 years • Teachers	• Program developed as curriculum to be implemented in the classroom. • Mediated by teachers.
**Estimulando funções executivas em sala de aula: o Programa Heróis da Mente (no prelo)** Carvalho & Abreu, 2014 (Brazil)	• To stimulate cognitive domains such as attention, memory, self-regulation and planning.	• Preschool children • Teachers	• Program developed as curriculum to be implemented in the classroom. • Mediated by teachers.
**Neuroeduca - Inserção da neurobiologia na educação ** Guerra, Pereira, & Lopes, 2004 (Brazil)	• To guide education professionals to use the knowledge of neuroscience in education and the approach to learning problems.	• Early Childhood Education Teachers • Caregivers of educational centers. • Elementary School Teachers (1st to 8th grade).	• - Teachers are the target of the intervention. **Steps:** • Identification of demand of the teachers. • Discussion about neurobiological foundations of learning process. • Interventions to improve the learning process.
**Desenvolvimento de habilidades metacognitivas: capacitação de professores do Ensino Fundamental **Busnello, Jou & Sperb, 2012 (Brazil)	• To promote the training of teachers for the development of cognitive, metacognitive and motivational learning in elementary school students.	• Students and teachers of the 5th grade of elementary school.	• Teachers are the target of the intervention. **Steps:** • Theoretical foundations: metacognition, intelligence, selective attention, reading comprehension, emotion and motivation. • Neuropsychology of the learning process. • Learning difficulties. • Planning activities to develop cognitive and metacognitive strategies with students.

The contents and materials involved in the psychoeducation and training program were drawn from the review of MEC parameters and school curricula. As a result, the CENA was structured around the following objectives:

[a] general primary education goals; and
[b] goals and contents of the Portuguese and Mathematics curricula for grades 1 and 2.

Primary education goals include the development of the following skills:

[a] the ability to take a critical, responsible and active stance in situations requiring conflict mediation and decision making;
[b] the perception of oneself as an agent of change, attentive to the elements in the environment and the interaction between them;
[c] the use of different forms of language to produce, express and communicate ideas; and
[d] the ability to question, as well as formulate and solve problems, using logic, creativity, intuition and critical thinking skills.

Additional achievements are listed in the Portuguese and Mathematics curricula. These include the following:

[a] to expand and adjust the use of spoken and written language to address different goals, topics and audiences;
[b] to comprehend oral and written text, as well as interpret and infer the intentions of others across different social contexts;
[c] to use language as a learning tool, knowing how to extract and use textual information (identify main points, take notes, list topics, produce coherent texts, outlines, and summaries);
[d] to perceive mathematics as a way to understand and transform the environment;
[e] to see intellectual work (mathematics) as an incentive for interest, curiosity, inquisitiveness and problem-solving;
[f] to establish relationships between quantitative and qualitative concepts using different types of mathematical knowledge (arithmetic, geometry, algebra); to use different logical and reasoning methods (deduction, induction, intuition) to solve problems and formulate results; and
[g] to describe, represent and present results in an accurate manner, discuss inferences, and make adequate use of oral language and its association with mathematical concepts.

The review of the national curriculum and teaching standards for grades 2 and 3, especially for Portuguese and Mathematics, also helped establish the goals and contents of the CENA Program. This information was used to teach participants about the cognitive functions underlying different school activities, and help teachers make more effective use of available resources to stimulate EF in the classroom. The review of curricular contents and activities also helped define the role of the teachers in the CENA Program, as did the education expert who contributed to the planning and structuring of the intervention.

**Program development - author brainstorming.** The structure of the CENA Program was based on a group cognitive-behavioral therapy (GCBT) approach involving the use of modeling.^42^ The intervention took place over^11^ a series of four-hour sessions held every other week. The first two sessions provided a theoretical introduction to neuropsychology and education, as well as the cognitive constructs addressed in the program (attention, memory, language and the EF, including self-regulation and metacognition).

The following two meetings involved an in-depth study of each cognitive skill targeted in the intervention (planning, inhibitory control, WM and cognitive flexibility). This stage was divided into modules corresponding to each target component. Each module, in turn, involved the introduction of several stimulation strategies of increasing complexity targeting the cognitive skill in question. As such, the contents of each module, as well as the order of the modules themselves, were organized based on a hierarchy of cognitive complexity. The logical sequencing task - organizing ideas and information, for instance, was less complex than the turning the story backwards task, and was therefore introduced earlier. The latter task consisted of a series of activities involving story-telling and repetition, which required several executive skills such as organization, planning, WM and cognitive flexibility.

The aim of these activities was to provide teachers with a first-hand opportunity to see how classroom activities could be used to stimulate the EF, especially those involved in the achievement of educational goals and learning objectives. Some of the tasks are very well known, and similar to activities which are freely available on the internet. Table 3 shows the list of activities developed in the program and the ideas that inspired their development.

**Table 3:** List of activities and sources consulted for stimulation of EF in the school setting.

Module / Sessions	Activities	Sources inspiring activities
Planning 3^rd^ and 4^th^ session	**1.** Organization of school backpack **2.** Organization of classroom materials **3. ** Homework organization **4.** Organization of a school diary **5.** Organization of ideas and information - logical sequencing **6.** Organization of ideas and information - storytelling and retelling stories **7.** Inventing a story - Part 1 **8. ** Inventing a story - Part 2	**1, 2, 3 and 4.** Activities of the school routine, redrafted for this program **5.** Subtest "figures arrangement" from WISC-III **6, 7 and 8.** Activities of reading and interpreting - teaching material (progressive increase in complexity)
Inhibitory control 5^th^ and 6^th^ session	**1.** Play called "Statue" **2. ** Play called "Musical chairs" **3.** Game sounds and movements: "Dancing to the music" **4.** Play called "What is the opposite" **5.** Cancellation Activities **6.** Turning the paths Activities **7.** Sometimes yes, sometimes no: visual mode **8.** Sometimes yes, sometimes no: auditory modality **9.** Mind what you say	**1,2 and 3.** Children's Play adapted for the program **4.** Sites games/children's activities **5. ** Cancellation Tasks (neuropsychological tests) **6.** Trail Making Test (2 levels of complexity) **7.** Go/no-go Paradigm (visual) (2 levels of complexity) **8.** Go/no-go Paradigm (auditory) (2 levels of complexity) **9.** Stroop Activities (2 complexity levels)
Working memory 7^th^ and 8^th^ session	**1.** Time for storytelling - creating a different ending **2.** Memory Game: words **3.** Memory Game: math operations **4.** Activity "what comes before" - visual mode **5.** Activity "What comes before" - auditory mode **6.** Activity "What are the words?" **7. ** Joining the dots - geometric figures **8.** Word Hunting Activity **9.** Numbers Hunting Activity - mathematical operations **10.** Domino game - mathematical operations	**1.** Activities of text reading and interpreting - teaching material (greater level of complexity) **2 and 3. ** Activities developed for this program **4 and 5.** N-back paradigms 1 and 2 - visual and auditory (2 levels of complexity) **6. ** Span of words in sentences auditory task - Neuspilin **7. ** Sites game/children's activities **8, 9 and 10.** Activities developed for this program
Cognitive flexibility 9^th^ and 10^th^ session	**1.** Activity: Creating figures **2. ** Categorization Activity **3.** Mind games **4. ** "And now ... what to do?" (Resolution of conflictive situations of everyday life) **5.** Retelling stories: new ending for the characters **6.** Turning the story backwards **7.** A different game (balloons) **8.** Activity: understanding the figures **9.** Activity: a trip to the desert **10. ** Activity: finding the color **11.** Hangman **12. ** Tangram **13.** Each figure in its place **14. ** Hidden Word	**1.** Five point test **2. ** Activity developed for this program **3.** Sites games/children's activities **4.** Activity developed for this program **5 and 6.** Activities developed for this program (progressive increase in complexity) **7.** Activity developed for this program **8 and 9.** Activities based on the Wisconsin Card Sorting Test **10.** Game sites/children's activities **11.** Teaching materials **12.** Sites games / children's activities **13.** Activity developed for this program **14.** Teaching materials

Each session began with a discussion of the contents introduced in the previous meeting, and ended with an oral and written assessment of the session. A questionnaire was developed for teachers to provide their opinion on the topics discussed and activities performed in each meeting. Additional details regarding the contents of each session are described in Pureza & Fonseca (2016).

**Evaluation by expert judges.** The inter-rater agreement (CVI) on the adequacy of the structure (language and methods) of the CENA Program, and its ability to achieve its intended goals, was 1.0. Although judges achieved an agreement rate of 100%, they also provided suggestions for improvement. These included the order in which cognitive skills were presented in the introduction section (attention, language, memory and EF), and the need for clearer and more specific instructions for the practical activities.

A selection of twenty such tasks was rated by three judges, who also reached 100% agreement with regards to their content validity. Slight changes were then made to the program to accommodate the following suggestions:

[a] providing more specific distinction between the difficulty levels of some activities, especially the connecting paths task;
[b] implementing some of the activities, such as organizing the classroom, during the actual sessions;
[c] improving the clarity and objectivity of task instructions.

## DISCUSSION

The construction of the CENA followed all methodological guidelines for the construction and/or adaptation of assessment instruments. Three stages in its development were especially important, namely:

[a] the review of existing teacher training programs for the stimulation of EF in school-age children;
[b] the examination of national curricula and school syllabi; and
[c] brainstorming and expert judge analyses.

The theoretical background for the CENA Program was drawn from the literature on the impact of stimulating the EF on childhood development and learning.^12,25-27,29^ The examination of MEC parameters and school curricula allowed the contents of the intervention to be adjusted to the educational goals of the targeted school years.

According to the literature, classroom activities developed by teachers can stimulate childhood cognitive development.^21^ We hope that the ideas and insights inspired by the CENA Program contribute to the development of creative and systematic strategies for the stimulation of EF in schoolchildren. Teachers often become responsible for the stimulation of EF and other cognitive skills in children as a result of schooling. The main difference between the CENA and other neuropsychological programs for teachers is the fact that this intervention focuses specifically on psychoeducation regarding student cognitive stimulation.

The majority of existing interventions focus on the stimulation of EF and self-regulation in preschool children, with teachers taking on the role of mediators.^25,26^ The only exceptions appear to be the Sarilhos do Amarelo program, which targets children aged 7 to 10, and the metacognitive stimulation program, which was developed for grade 5 students.^28,41^ Although the majority of interventions described in the literature target preschool children,^25,26,29,40^ they still provided important theoretical contributions to the development of the CENA Program.

A methodological assessment of existing interventions also revealed a lack of published studies on the stages involved in the construction of these programs. The PIAFEX appears to be the only intervention for which detailed construction data and validity evidence is available.^43^

Methodological guidelines are essential for the development of assessment and/or intervention tools, and crucial for the verification of constructs such as content validity and reliability.^31,33,39,31,44^ The inter-rater analysis performed in the present study allowed for an assessment of the congruence between the main objectives of the CENA Program and its structure, language and methods, as well as the extent to which the intended goals were achieved by the intervention. The high levels of inter-rater agreement (CVI – 1.0) also provided important evidence of content validity for the CENA, confirming the contribution of methods and strategies drawn from neuropsychological assessment to the field of cognitive intervention. Methodological investment is essential to obtain future evidence of the applicability, efficacy and effectiveness of an intervention.

The CENA was structured according to a GCBT model.^42^ This format was selected to provide an opportunity for participants to discuss and develop their own strategies, and allow for observation-based modeling.^42,45^ Group interventions also benefit a large number of participants in a short period of time and at a lower cost (since few individuals are required to train several others), in addition to being more easily replicable and having lower infrastructure demands.^42^

The CENA Program was structured to contain both a general theoretical introduction to educational neuropsychology and cognitive abilities, as well as opportunities for the discussion of individual cognitive components. Each session began with a discussion of the topics introduced in the previous meeting, and ended with a final discussion which summarized psychoeducational contents and provided a systematic opportunity for the assessment of session contents and activities by the teachers.

In light of the importance of educational interventions for the cognitive stimulation of school-age children, future studies should examine the effectiveness of teacher training programs for the stimulation of EF in typically developing children across different age groups. These studies may contribute to the development of interventions which address more specific educational demands, such as reading, writing and mathematical difficulties, as well as neurodevelopmental conditions such as ADHD and communication disorders. In conclusion, teacher training in school neuropsychology may constitute an important preventive policy measure, which could contribute to academic development and educational attainment in Brazil, paving the way for progressive improvements and solid education.
